# The efficacy of a porcine collagen matrix in keratinized mucosa width augmentation: a 10-year follow-up clinical prospective study

**DOI:** 10.1186/s40729-023-00475-1

**Published:** 2023-04-12

**Authors:** Mattia Manfredini, Pier Paolo Poli, Pierluigi Guerrieri, Mario Beretta, Carlo Maiorana

**Affiliations:** 1 Implant Center for Edentulism and Jawbone Atrophies, Maxillo-Facial Surgery and Dental Unit, Fondazione IRCCS Cà Granda Ospedale Maggiore Policlinico, 20122 Milan, Italy; 2 Department of Biomedical, Surgical and Dental Sciences, University of Milan, Via Della Commenda 10, 20122 Milan, Italy

**Keywords:** Collagen matrix, Soft tissue augmentation, Keratinized mucosa, Peri-implant mucosa, Peri-implant soft tissue, Clinical prospective study

## Abstract

**Purpose:**

The aim of the present study was to evaluate the long-term effectiveness of a xenogeneic collagen matrix (CM) in pre-prosthetic augmentation of the keratinized mucosa width (KMW) at implant sites.

**Methods:**

All of the patients with localized implant-supported rehabilitation previously treated with vestibuloplasty and KMW augmentation with a non-cross-linked porcine CM were recalled. KMW was measured clinically in an apico-coronal direction from the mucogingival junction to the mucosal margin at the prosthetic zenith of each crown. Measurements were performed clinically by means of a PCP-UNC15 periodontal probe and rounded to the nearest mm. KMW values recorded at 1 year, 5 and 10 years from the soft tissue augmentation procedure were compared using a one-way repeated-measures ANOVA with Bonferroni multiple comparison post-hoc analysis. The level of significance was set at 0.05.

**Results:**

Measurements were conducted on 13 patients. All implants were in function with no signs of biological complications. All except one implant site showed KMW ≥ 2 mm. KMW decreased significantly from 1 year (3.33 ± 1.11 mm) to 5 years (2.77 ± 0.92 mm) (*p* = 0.001), and finally remained stable from 5 to 10 years (3.2 ± 0.99 mm) (*p* = 0.607). From a visual aspect, peri-implant soft tissues were characterized by a good texture and color blending compared to the adjacent teeth, highlighting good integration of the remodeled tissues and stability of the esthetic result.

**Conclusions:**

The use of a CM in pre-prosthetic soft tissue augmentation at implant sites has proven to be effective in obtaining and maintaining at least 2 mm of KMW around dental implants over a follow-up of 10 years.

## Background

Placement of dental implants in edentulous patients is regarded as a safe and predictable long-term therapy [1]. Different variables play a major role in the long-term aesthetic and functional success of an implant-supported rehabilitation. Among them, adequate volume and stability of peri-implant soft tissues are essential to maintain the result stable [2]. Considering the peri-implant phenotype, keratinized mucosa width (KMW) represents the height of the keratinized soft tissue in the apico-coronal direction from the mucogingival junction to the margin of the peri-implant mucosa [3].

The need for an adequate peri-implant KMW, both for functional and aesthetic reasons, has been widely discussed in the literature. According to the ‘2017 World Workshop on the Classification of Periodontal and Peri-Implant Diseases and Conditions’, the evidence regarding the effects of the presence or absence of keratinized mucosa (KM) on the long-term health of peri-implant tissues is equivocal and limited [4]. For some, the evidence is weak regarding a direct correlation between inadequate KMW and increased risk of implant failures, mucosal recessions [5, 6] and the development of peri-implantitis [7]. Conversely, soft tissue deficiency around implants (KMW < 2 mm) has been associated with increased plaque accumulation, increased mucosal inflammation [8–13], higher probing depth, increased tendency to soft tissue recession and clinical attachment loss [14]. Accordingly, a minimum of 2 mm of KMW has been shown to minimize the incidence of peri-implant mucositis and marginal bone loss in poorly compliant patients [15]. This protective effect has been observed also in the presence of plaque-associated inflammation [15]. Other advantages have been linked to an adequate quality and quantity of KMW, including greater plaque control by patients and better resistance to brushing trauma [16], together with a better aesthetic result [16, 17].

The KM may sometimes be extremely poor or even absent. In these circumstances, the most coronal part of peri-implant soft tissues adjacent to the prosthetic crown is represented by alveolar mucosa [7]. In this scenario, soft tissue augmentation procedures are necessary to increase peri-implant KMW, taking into consideration that mucogingival procedures around implants are more challenging than at teeth, due to the absence of the periodontal ligament [18]. Surgical procedures advocated to optimize peri-implant soft tissues can be categorized into techniques used to increase the width or the thickness of the KM. The former include free gingival grafts (FGGs), apically positioned flap (APF), or APF and vestibuloplasty [19, 20], eventually associated with a soft tissue graft [19]. In 1962, Friedman [21] proposed the term ‘apically repositioned flap’ to more appropriately describe the technique introduced by Nabers [22] in 1954. Since then, the apical repositioning flap has been successfully used to increase the width of attached gingiva around natural teeth [23]. This technique has been subsequently adapted to increase the KMW around implants as an alternative to FGGs [24–26]. The advantages of APF compared to FGG are a low post-operative morbidity due to the absence of a donor site and a greater aesthetic integration and color blending, which make this technique preferable in the upper arch [24]. Furthermore, since APF implies by definition a displacement of the mucogingival junction, it is particularly indicated after guided bone regeneration techniques, where, to obtain a tension-free flap closure, passivation of the flaps is needed to allow their coronal displacement, leading to a subversion of the mucogingival architecture [27]. Given that the mucogingival line tends to relocate to the original and genetically determined position after a few years [28–31], one of the aim of the APF is precisely to restore its initial level. At the same time, vestibuloplasty techniques can generally be classified as submucosal (for mucosal advancement), vestibuloplasty for secondary epithelialization, and vestibuloplasty combined with soft tissue grafts [20]. The goal of such procedures is to create an adequate depth of the fornix and limit the traction of fibers and muscle insertions [32, 33].

Over the years, autogenous soft tissue grafts and soft tissue substitutes have been proposed and studied to optimize peri-implant soft tissues [34]. Despite the results obtained with the latter, autografts are currently considered the gold standard [19]. Nevertheless, soft tissue substitutes have been developed to offer comparable performance to autogenous tissue and overcome its limits, such as the limited availability and the post-operative patient discomfort [35]. In the last decades, several non-autologous materials have been evaluated to increase peri-implant KM [36, 37]. Soft tissue substitutes have proven to be effective alternatives to FGG [38–43], but, on the other hand, they have shown a high resorption rate over time [44]. Among the most used soft tissue substitutes, acellular dermal matrices (ADMs) and collagen matrices (CMs) can be included, the latter being further distinguished in cross-linked and non-cross-linked. One of the most documented cross-linked CM is composed of type I and III collagen extracted from certified pigs subjected to pre- and post-mortem health tests before processing. The collagen is then chemically purified to break down the antigenic power and finally, after packaging, the matrix is sterilized by gamma-irradiation to inactivate microorganisms and viruses. Cross-linked CM are porous and volumetrically stable, because the collagen is subjected to a chemical cross-linking process. These features support the stabilization of the blood clot, its cellular colonization and angiogenesis [45, 46]. Non-cross-linked CMs contain type I and III collagen. The entire production process, similar to the cross-linked CM, results in a stable three-dimensional matrix consisting of collagen and elastin without further cross-linking or chemical treatment [47, 48]. In general, compared to autologous tissue grafts, soft tissue substitutes offer several advantages including theoretical unlimited amount, ease of use and handling, greater esthetic integration, shortened operative times, the absence of a donor site and related morbidity, reduction of post-operative discomfort and consequent painkillers intake, as well as greater overall patient satisfaction [19, 24, 49–54].

As mentioned above, many studies have investigated the mechanical and biological properties of soft tissue substitutes, and in particular CMs. However, to the best of the authors’ knowledge, the evidence about long-term data concerning the stability of the results obtained with such substitutes in peri-implant soft tissue augmentation is extremely poor. Thus, the aim of this work was to evaluate the effectiveness of a porcine collagen non-cross-linked CM in maintaining the augmented KMW stable around dental implants over a 10-year period.

## Materials and methods

### Study design/setting

The study was designed as a prospective observational non-controlled clinical study. All of the procedures were performed according to the ethical principles for medical research involving human subjects outlined in the World Medical Association Declaration of Helsinki. The study protocol has been reviewed and approved by the Ethics Committee of the Fondazione IRCCS Cà Granda, Ospedale Maggiore Policlinico, Milan, Italy (Area 2, study No. 10826-01). Surgical, prosthetic and follow-up procedures were explained in detail, and each patient signed an informed consent. The soft tissue substitute used in the study consisted in a non-cross-linked CM (Mucograft^®^, Geistlich Pharma AG, Wolhusen, Switzerland) made of pure collagen type I and III matrix of porcine origin without further cross-linking. The said CM has a double-layer structure: a thin, smooth and low-porosity compact layer, namely, the side facing the oral cavity, composed of compact collagen able to promote the marginal adaptation and protection of the deepest layer. This surface layer has a smooth and elastic conformation to allow easy handling during suture maneuvers. The second layer consists of collagen that is more porous, thicker and three-dimensional spongy than the first layer. This rough surface is positioned on the underlying connective tissue and allows tissue integration through the facilitation of blood clot formation and promoting neoangiogenesis.

### Inclusion/exclusion criteria

All patients previously treated with soft tissue augmentation procedures by means of non-cross-linked porcine CM were recalled for the 10-year follow-up visit. Detailed inclusion and exclusion criteria were listed in the first article [53]. In brief, patients initially included were partially edentulous female and male subjects with at least 18 years of age, both systemically and periodontally healthy, with a good oral hygiene, requiring pre-prosthetic localized soft tissue augmentation procedures at implant sites. Smoking patients (> 10 cigarettes/day) and patients presenting with local or uncontrolled systemic diseases that could influence bone turnover/wound healing were excluded.

### Treatment

The surgical procedures performed in this work were already described in previous studies [39, 53] and consisted in a combination of APF and vestibuloplasty. In brief, a split-thickness longitudinal incision in the residual KM associated with vertical releasing incisions placed mesially and distally to the treated area were made using a 15C blade (Henry Schein Inc., Melville, NY, USA). A periosteal bed was prepared, and any muscle fiber, fibrous banding and submucosal fatty tissue were gently detached. The apical portion of the mucosal flap was anchored to the periosteum in the depth of vestibule. At this point, a CM (Geistlich Mucograft^®^, Geistlich Pharma AG, 6110 Wolhusen, CH) adapted to the surgical area was sutured to the perimeter of the periosteal bed with 5–0 nylon single stitches (Ethilon, Ethicon GmbH, Norderstedt, Germany). In this way, the denuded area of the wound in the vestibule was entirely covered by the CM. On the day of surgery, an acrylic splint was placed over the vestibuloplasty site to protect the grafted area, and was left in place for 10 days. During this period, patients were asked to irrigate with 0.9% NaCl solution and rinse with 0.2% chlorhexidine (Corsodyl, Glaxo-SmithKline, Brentford, UK). In all cases, dental implants were placed after a healing period of 2 months. The re-entry surgery to place the healing abutments was performed after 4 months in the maxilla and 3 months in the mandible. Definitive prostheses were finally delivered 1 month thereafter. All patients were enrolled in a supportive maintenance therapy program tailored on the basis of the patient compliance. At least 2 professional oral hygiene sessions were scheduled per year.

### Study endpoint

**Fig. 1:**
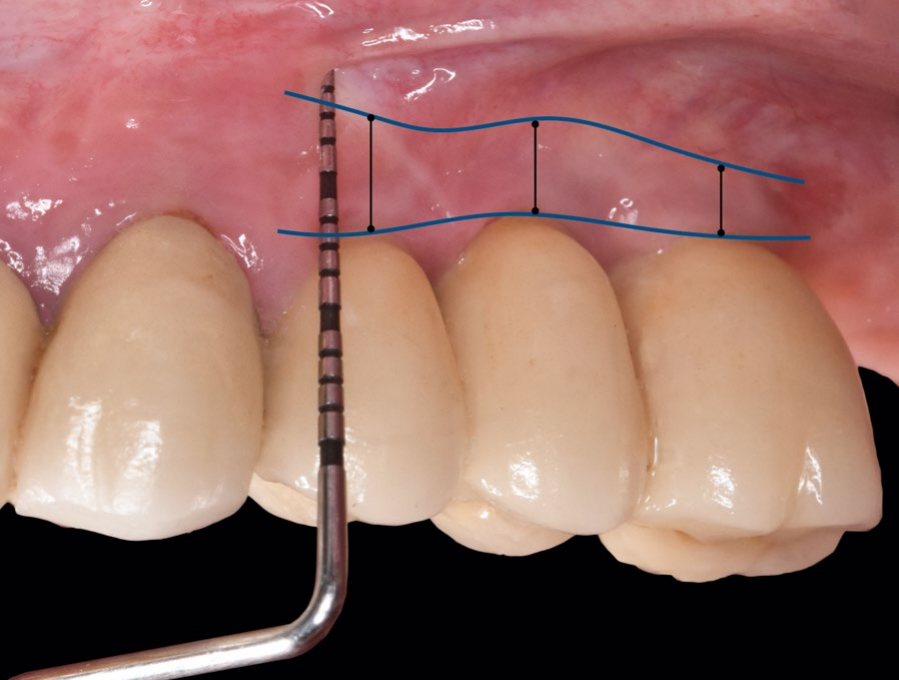
Clinical intraoral image illustrating KMW measurements performed 1 year, 5 years and 10 years after the soft tissue augmentation procedure. The black lines indicate, at each implant site, the KMW measured from the mucogingival junction to the peri-implant mucosa margin at the prosthetic zenith of each implant crown

In the present 10-year follow-up study, the endpoint was the stability evaluation of the KMW over time, defined as the height of the KM in the apico-coronal direction from the mucogingival junction to the margin of the peri-implant mucosa [3]. To fulfil this aim, all patients initially enrolled were recalled for a follow-up examination. Apico-coronal measurements were registered clinically at each treated site using a PCP-UNC15 periodontal probe (Hu-Friedy, Chicago, IL, USA) rounded to the nearest millimeter. To compare the KMW registered at 1 year and 5 years from the surgical procedure, the same reference points used in the 5-year study were used [39]. In particular, the prosthetic zenith at each implant crown was used to replicate the same position of the measurement during the study periods. Thus, the KMW was measured from the mucogingival junction to the peri-implant mucosa margin at the prosthetic zenith of each implant crown (Fig. 1).

### Statistical analysis

Statistical analyses were performed using IBM SPSS Statistics 24.0 (IBM Corp, Armonk, NY), adopting the surgical site as the statistical unit. Descriptive statistics of the measured continuous values were used to explore the KMW in the cohort of patients during the different time intervals, including mean ± standard deviation and shrinkage percentage. The mean KMW measured at each site and expressed in millimeters was considered in the statistical analysis. The Shapiro–Wilk test was used to assess the normality of data distribution. Because the distribution of the data met the requirements for normality and homogeneity of variance assumptions (*p* > 0.05), quantitative data were analyzed using parametric tests. In particular, one-way repeated-measures analysis of variance (ANOVA) with Bonferroni multiple comparison post-hoc analysis was conducted to evaluate the KMW variation among the study intervals. The level of significance was set at 0.05.

## Results

**Fig. 2:**
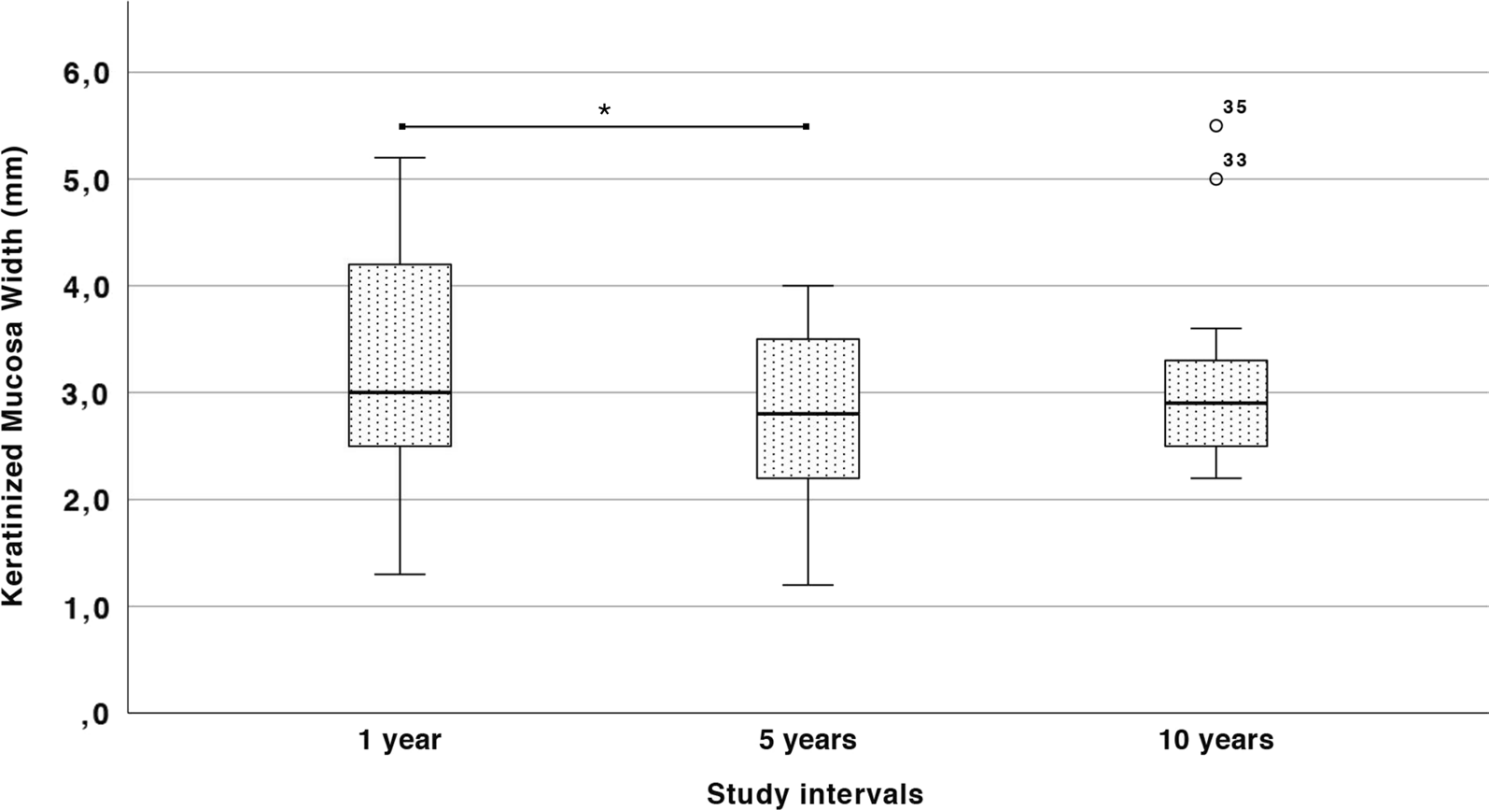
Box plot illustrating the KMW trend during the study intervals. * indicates statistically significant differences (*p* < 0.05)

Originally, a total of 15 patients were enrolled, consisting of 12 females and 3 males, aged between 43 and 72 years at the time of inclusion. Of them, 11 patients were rehabilitated in the mandible, while 4 in the maxilla. A total of 33 rough-surfaced fixtures were placed supporting 15 implant-supported rehabilitations (Table 1). No early post-operative complications were observed and the healing proceeded uneventfully for all patients. At the 1-year follow-up, 2 drop outs were registered due to biological complications at the augmented sites. No further complications were observed. The remaining 13 patients were recalled for the clinical evaluation of the KMW at the 10-year follow-up. All implants were in function with no signs of biological complications. All except one implant site showed KMW ≥ 2 mm. The repeated measures ANOVA determined that mean KMW differed statistically significantly between the study intervals (*p* < 0.01) (Fig. 2). Post-hoc analysis with a Bonferroni adjustment revealed that KMW decreased significantly from 1 year (3.33 ± 1.11 mm) to 5 years (2.77 ± 0.92 mm) (*p* = 0.001), and finally remained stable from 5 to 10 years (3.2 ± 0.99 mm) (*p* = 0.607). From a visual aspect, peri-implant soft tissues were characterized by a good texture and color blending compared to the adjacent teeth, highlighting good integration of the remodeled tissues and stability of the esthetic result. Considering 1 year from the surgical phase as the baseline study period, overall graft contraction was 16.81% at 5 years and 3.9% at 10 years (Table 2).

**Table 1:** Demographic characteristics of the treated sites

Patient ID	Age	Gender	Implants	Replaced teeth
101	54	F	2	45–46–47
102	82	M	3	14–15–16
103	74	F	2	36–37
104	69	F	2	24–25–26
105	62	F	2	35–36
106	82	F	2	14–15–16
107	55	M	2	16–17
108	64	F	2	42–41–31–32
109	58	F	2	34, 36
110	57	F	1	46
111*	55	F	4	34–35–36–37
112	72	M	2	15–16
113*	53	F	2	35–36
114	57	F	3	35–36–37
115	71	F	2	44–46

*Patients dropped out from the study due to biological complications before the 1-year follow-up examination. Patient 111 experienced a peri-implantitis that was solved with a conventional free gingival graft procedure; Patient 113 developed a peri-implant mucositis with profuse bleeding on probing

**Table 2:** Mean KMW values measured at each treated site

Patient ID	Mean KT width (mm)					Shrinkage (%)	
	Pre-op	Post-op	1 year	5 years	10 years	5 years	10 years
101	0	6,4	4	3,5	2,6	12,5	35
102	0	4,6	1,3	1,2	1,5	7,69	− 15,38
103	0,3	6,7	3	2,8	2,8	6,67	6,67
104	0	6,5	2,5	2,2	2,8	12	− 12
105	1,3	6,4	3,8	3,3	3,6	13,16	5,26
106	1,2	6,8	4,5	4	2,2	11,11	51,11
107	0	7	5,2	4	5	23,08	3,85
108	0,5	7,3	4,2	3,8	3	9,52	28,57
109	1,1	5,5	4,5	3	5,5	33,33	− 22,22
110	0	6,3	2,6	2,3	3,3	11,54	− 26,92
112	1,3	5	3	2,5	3	16,67	0
114	0	5,5	2,3	2	3	13,04	− 30,43
115	0,2	4,4	2,5	1,5	2,5	40	0

## Discussion

The purpose of the present study was to evaluate the long-term efficacy of a porcine derived non-cross-linked CM used as graft material in combination with vestibuloplasty/APF techniques in maintaining KMW around dental implants. To this end, measurements registered in the present 10-year study were compared to those recorded at 1 year and 5 years following soft tissue augmentation. The rationale was to replicate the measurements using the same reference points used throughout the entire follow-up period. To fulfil this aim, the zenith of the prosthetic crowns and the mucogingival junction were used as fixed reference landmarks in the clinical evaluation of the KMW. The results obtained herein showed a certain stability of the KMW from 5 to 10 years, while a slight contraction has been observed from 1 to 5 years. It is worth noting that mean KMW values remained > 2 mm during the entire study period, with only one implant site showing less than 2 mm of KMW at 10 years.

Optimization of peri-implant soft tissues is generally indicated in case of insufficient quantity and/or quality of KM, responsible for aesthetic and functional imperfections. Current literature considers autogenous soft tissue as the gold standard material, with the palate as the most frequent intraoral donor site, regardless of the surgical approach [19]. A donor site, however, greatly increases patient discomfort during the healing phases, with the corono-apical size of the graft and the thickness of the residual soft tissue being two important variables impacting post-operative pain [35]. Moreover, presence of a donor site necessarily entails an increase in the likelihood of complications. A recent split-mouth study compared the risk of intra- and post-operative bleeding by evaluating different palatal harvesting techniques [55]. The results highlighted how the trap door technique can significantly increase bleeding complications, due to a greater risk of damaging the major vessels during the harvesting procedure, which in turn leads to greater post-operative discomfort of the patient. It is also worthy of note that, rather than the procedure itself, post-operative bleeding might be more associated with post-surgical trauma and irritation of the surgical site during the stomatognathic functions [49]. In this respect, the use of CMs allows avoiding donor sites in the palate, which in turn may decrease the post-operative patient morbidity considerably. In this respect, the cohort of patients treated in the present study reported no bleeding during the immediate post-operative course, highlighting the good hemostatic effect of the CM and its capability to promote blood clot formation [53]. The fact that most of the treated patients did not feel any pain at all, except 2 patients that took a mild analgesic after surgery, corroborates the use of CMs to reduce post-operative patient morbidity [53].

Apart from the limited availability, another disadvantage frequently correlated with the use of autogenous soft tissue grafts is the mismatch in color and texture compared to the adjacent tissues [56, 57]. In this matter, the grafted areas observed in the present study after 10 years showed good color blending, without any dyschromia with the surrounding tissue, providing a satisfying esthetic integration. This may be considered an improvement compared to standard protocols, where FGGs retain the native appearance of the hard palate, and the color matches poorly with the surrounding tissue, where the graft is positioned [34, 58]. It should also be noted that, differently from FGGs that are grafted with the epithelial portion, acellular CMs are epithelialized by proliferation of the surrounding tissue at later stages. This may have an additional positive effect in the esthetic integration of the CM in contact with the host KM, leading to better tissue texture and color match to surrounding native tissues.

Concerning the surgical technique, a recent systematic review by Tavelli et al. [57] showed a significant increase in KMW when APF was combined with a graft material, whether autogenous or soft tissue substitute, while no statistically significant gain was obtained following any of the bilaminar techniques. Interestingly, Monje et al. [59] found that FGGs undergo a shrinkage in the original size of about 40% at 6 months, with an additional 10% contraction at implant sites. Similarly, another study showed a shrinkage of FGGs of roughly 1.7 times at implant sites after 1 year [60], emphasizing the fact that even autogenous grafts may suffer KMW reduction over time, particularly around dental implants. In this respect, many studies evaluated the capability of CMs to gain KMW following peri-implant soft tissue augmentation. Sanz et al. [37] found that CM attained a mean KMW of 2.5 mm at 6 months. One year later, Lee et al. obtained a main value of 3.2 mm [61]. Similarly, Lorenzo et al. [62] observed a KMW of 2.80 mm with CMs, comparably to another study by Huang et al. [63]. Vellis et al. [64] found a higher increase of KMW compared to the previously mentioned studies, with a mean value of 4.4 mm. In a work by Jiang et al. [25], the mean augmented KMW was 4.81 mm after 3 months of healing. Overall, the results reported in these studies favourably comply with those reported herein, where a mean KMW of 3.33 ± 1.11 mm has been measured. At 5 years, the mean KMW obtained following soft tissue augmentation with CMs in the present study was 2.77 ± 0.92 mm. From a mathematical aspect, the difference was statistically significant, but from a clinical standpoint, KMW remained > 2 mm, still yielding a positive outcome. A remarkable amount of KMW has been observed by Schmitt et al. after 5 years from vestibuloplasty associated with CM grafting [38]. With the same CM employed in the present study, the authors registered a KMW of 6.15 ± 1.23 mm after 5 years, which is higher than that reported herein. It should be noted, however, that KMW was evaluated also in fully edentulous subjects who probably required extensive vestibuloplasty procedures in the entire rehabilitated area, a fact that might have increased the amount of KMW gained compared to the localized defect treated in the present study. Furthermore, at 5 years, the authors reported data of only 5 patients, which may have wakened the reliability of the long-term results. Nonetheless, after 10 years from the surgical procedure, the 13 patients recalled for the follow-up examination in the present study showed a mean KMW of 3.2 ± 0.99 mm, without any statistically significant difference compared to the 5-year data, emphasizing the stability of the result on the long term. Moreover, the remodelling rate of the KMW observed during the 10-year follow-up confirms the assumption that the height of KM is genetically pre-determined and the mucogingival junction tends to relocate to the original position [28–30]. Indeed, KMW was never lower than that observed at the adjacent natural elements and, after an initial remarkable contraction during the first year, remained stable, > 2 mm, during the entire follow-up period.

Some limitations should be taken into consideration when interpreting these results. First of all, no sample size calculation has been performed, leading to a small sample of patient. It should be noticed, however, that some of the previously mentioned studies using xenogeneic CMs have been published with even less participants and with reduced follow-up times, corroborating the results found in this investigation and making them comparable with those reported in the current literature. Another limitation is related to the absence of a control group, giving the fact that FGG is still considered the gold standard to increase KMW. However, despite the different amount of KMW that can be achieved with either FGGs or soft tissue substitutes, it seems that CMs may provide a sufficient height of peri-implant KM that, most importantly, remains stable on the long term. Notwithstanding, all of these concerns recognize a lack of external validity and demand that the reported results should be interpreted with caution and should not be extrapolated to the general population.

## Conclusions

Within the limitation of the present study, the use of a porcine CM in pre-prosthetic KMW augmentation yielded favorable results on the long term. Following initial remodeling, the KMW remained substantially stable and > 2 mm from 1 to 10 years after soft tissue augmentation procedure.

## Data Availability

Data will be made available upon reasonable request by the corresponding author of the manuscript.

## Abbreviations

KM: Keratinized mucosa
KMW: Keratinized mucosa width
CM: Collagen matrix
ADM: Acellular dermal matrix
APF: Apically positioned flap
FGG: Free gingival graft
